# Asymmetric Sulfoxidations Catalyzed by Bacterial Flavin-Containing Monooxygenases

**DOI:** 10.3390/molecules29153474

**Published:** 2024-07-25

**Authors:** Gonzalo de Gonzalo, Juan M. Coto-Cid, Nikola Lončar, Marco W. Fraaije

**Affiliations:** 1 Departamento de Química Orgánica, Universidad de Sevilla, c/Profesor García González 1, 41012 Sevilla, Spain; jcoto@us.es; 2 Gecco Biotech B.V., Zernikepark 6-8, 9747AN Groningen, The Netherlands; n.loncar@gecco-biotech.com; 3 Molecular Enzymology Group, Groningen Biomolecular Sciences and Biotechnology Institute, University of Groningen, Nijenborgh 4, 9747AG Groningen, The Netherlands; m.w.fraaije@rug.nl

**Keywords:** biocatalysis, chiral sulfoxides, flavin monooxygenases, asymmetric oxidations

## Abstract

Flavin-containing monooxygenase from *Methylophaga* sp. (*m*FMO) was previously discovered to be a valuable biocatalyst used to convert small amines, such as trimethylamine, and various indoles. As FMOs are also known to act on sulfides, we explored *m*FMO and some mutants thereof for their ability to convert prochiral aromatic sulfides. We included a newly identified thermostable FMO obtained from the bacterium *Nitrincola lacisaponensis* (*Ni*FMO). The FMOs were found to be active with most tested sulfides, forming chiral sulfoxides with moderate-to-high enantioselectivity. Each enzyme variant exhibited a different enantioselective behavior. This shows that small changes in the substrate binding pocket of *m*FMO influence selectivity, representing a tunable biocatalyst for enantioselective sulfoxidations.

## 1. Introduction

Chiral sulfoxides are crucial in biology, serving as key intermediates in the synthesis of many pharmaceuticals. Their unique stereochemistry influences drug efficacy and safety, impacting interactions with biological targets, which is vital for producing specific therapeutic effects [1,2]. These molecules also serve as versatile building blocks and chiral auxiliaries, facilitating the construction of complex molecules with high stereocontrol. Additionally, they are pivotal in asymmetric catalysis, where they act as ligands for transition metals, promoting enantioselective transformations [3,4]. The preparation of optically active sulfoxides through the catalytic asymmetric oxidation of prochiral sulfides is a well-established methodology [5,6,7]. The use of biological systems (using whole cells or isolated enzymes) as catalysts for these reactions has gained an overwhelming interest in the last few years, due to the advantages that biocatalysis offers over classical methodologies [8,9,10]. Biocatalyzed oxidations are mediated by biodegradable, non-toxic and non-hazardous catalysts which require mild reaction conditions and molecular oxygen or hydrogen peroxide as mild oxidants [11]. Several distinct biocatalytic approaches for the preparation of chiral sulfoxides have been employed [12], including the use of peroxygenases [13], peroxidases [14,15] or monooxygenases [16,17,18,19,20]. The application utilizing monooxygenases is very attractive, as these enzymes can catalyze oxygenations by merely employing molecular oxygen as an oxidant. While one of the oxygen atoms is inserted in the substrate, the second one is reduced to water. Many monooxygenases contain a flavin cofactor as a prosthetic group [21]. These flavoprotein monooxygenases (FPMOs) can be divided into different classes according to structural and mechanistic properties. Class B FPMOs corresponding to monooxygenases are encoded by a single gene and contain a tightly bound FAD as cofactor [22]. They use NADPH as coenzyme. The so-called flavin-containing monooxygenases (FMOs, E.C. 1.14.13.8) are members of this class. FMOs can catalyze the selective oxygenation of different heteroatoms, including nitrogen and sulfur. Most of the scientific literature on FMOs concerns mammalian FMOs, as they are crucial in the detoxification (and sometimes the activation) of drugs and xenobiotics [23,24,25]. In 2003, the first bacterial FMO (from a *Methylophaga* sp. (*m*FMO)) was discovered, and was shown to be able to convert trimethylamine [26]. It attracted interest due to its ability to also convert indole and analogues into indigoid dyes. The crystal structure of this biocatalyst was solved in 2008, revealing the important role of the nicotinamide cofactor NADP^+^ in its structure and mechanism [27]. Two years later, *m*FMO was cloned and expressed in *E. coli* in sufficient amounts as a bifunctional biocatalyst, being fused with the NADPH-regenerating phosphite dehydrogenase from *Pseudomonas stuzteri* (PTDH) [28]. The fusion biocatalyst was employed in the enantioselective oxidation of a limited set of prochiral sulfides. In 2019, *m*FMO was engineered towards a better catalytic performance in producing indigo by using structure-inspired mutagenesis [29]. Modifications at the C78 and Y207 positions, as well as the triple mutant C78I/Y207W/W319A, showed higher thermostability and *k*_cat_ values for indole when compared with the wild-type enzyme. More recently, a novel bacterial thermostable FMO was obtained from the alkaliphilic bacterium *Nitrincola lacisaponensis* (*Ni*FMO) by a genome mining approach [30]. This biocatalyst can also be expressed in high amounts in *E. coli* and was shown to act on a wide range of typical FMO substrates.

In the present paper, the performance of *Ni*FMO, *m*FMO, as well as some of the *m*FMO mutants, were studied as biocatalysts for the sulfoxidation of different prochiral sulfides, with the aim of disclosing their ability to produce optically active sulfoxides.

## 2. Results

### 2.1. Comparision between mFMO and NiFMO in Biocatalysed Sulfoxidations

All biocatalysts utilized in this study have been expressed as fusion proteins, with phosphite dehydrogenase from *Pseudomonas stutzeri* serving as the N-terminal fusion partner. Phosphite dehydrogenase (PTDH) facilitates the efficient regeneration of NADPH, utilizing inexpensive phosphite as a sacrificial cosubstrate [31]. Initial investigations focused on evaluating the efficacy of *Ni*FMO for the enantioselective synthesis of various chiral sulfoxides. Comparative analysis with previously reported results for *m*FMO was conducted [28], excluding substrates **3–8a**, **14a**, **17a** and **18a**, which had not been tested with both FMOs (Table 1).

In general, *m*FMO exhibited higher activity compared to *Ni*FMO for nearly all examined substrates, as evidenced by the attained conversions. Notably, the oxidation of thioanisole (**1a**) yielded product (*S*)-**1b** with a high conversion and moderate enantiomeric excess (35%) when employing *m*FMO, while employing *Ni*FMO resulted in the (*R*)-sulfoxide, with significantly lower optical purity and conversion. Similarly, ethyl phenyl sulfoxide (**2b**) could be obtained with moderate-to-good enantiomeric excesses, with *m*FMO displaying greater selectivity than *Ni*FMO. Additionally, both biocatalysts yielded higher enantiomeric excesses for (*S*)-**2b** compared to its methyl counterpart. No sulfoxidation was observed for the vinyl derivative **3a** with either enzyme, while substrate **4a** was oxidized to the (*S*)-sulfoxide with low conversions and moderate optical purities by both biocatalysts. Incorporating a cyclic alkyl moiety in the sulfide structure, as in cyclopropyl phenyl sulfide **5a**, resulted in no oxidation by *Ni*FMO, with (*S*)-**5b** recovered with moderate conversion and low optical purity. Furthermore, the oxidation of other phenyl alkyl substrates containing heteroatoms in the alkyl chain (sulfides **6a** and **7a**) was catalyzed by *m*FMO with higher conversion, but lower optical purities compared to *Ni*FMO, allowing for the recovery of (*S*)-**6b** and (*S*)-**7b** with optical purities around 45% (entries 6 and 7).

Oxidation of thioanisole derivatives with varied substituents on the aromatic ring (entries 8–15) demonstrated superior performance of *m*FMO in terms of activity and selectivity compared to *Ni*FMO across most cases, notwithstanding differences in the electronic nature of the aromatic ring substituent. Notably, *p*-methyl (**10a**), *p*-chloro (**11a**), and *p*-bromothioanisole (**14a**) were oxidized to the corresponding (*S*)-sulfoxides with good conversions and optical purities around 90%, representing optimal substrates for *m*FMO. Substrates bearing strongly electron-donating substituents, such as *p*-hydroxy or *p*-methoxy, yielded sulfoxides (*S*)-**8b** and (S)-**9b** with enantiomeric excesses of 70%, while a strongly electron-withdrawing group, as in *p*-cyano, resulted in low optical purity for sulfoxide (*S*)-**15b**. Chlorothioanisole substitution patterns revealed that the *meta*-derivative produced the lowest optical purities (entry 12), while for *o*-chlorothioanisole, (*S*)-**13b** could be recovered with 64% enantiomeric excess, but only 20% conversion. Biooxidations catalyzed by *Ni*FMO followed a similar trend to *m*FMO but yielded lower optical purities for nearly all sulfides, except for substrate **15a**, which afforded (*S*)-**15b** with a higher optical purity (enantiomeric excess = 32%, entry 15). Oxidation of *p*-methyl, *p*-chloro and *p*-bromothioanisole yielded (S)-sulfoxides with enantiomeric excesses around 80%, with (*S*)-methyl *p*-methylphenyl sulfoxide (**10b***)* reaching a 97% conversion after 24 h at room temperature.

The *m*FMO demonstrated the ability to oxidize methyl naphthyl sulfide (**16a**) to (*S*)-**16b** with very low conversion and moderate optical purity, in contrast to *Ni*FMO, which exhibited no conversion with this compound. Neither FMO could catalyze the sulfoxidation of bulkier sulfides, including benzyl phenyl sulfide (**17a**) and pyrmetazole (**18a**), when operating at 5 mM substrate concentration and longer reaction times (entries 17 and 18).

Evaluation of benzyl alkyl sulfides (**19–20a**) revealed superior performance of *Ni*FMO compared to *m*FMO for methyl and ethyl derivatives, achieving higher enantioselectivities (entries 17 and 18). Notably, a reversal of enantiopreference was observed, with *m*FMO yielding (*S*)-**19**,**20b** with low optical purities (15–17% *ee*), while *Ni*FMO led to (*R*)-**19**,**20b** with enantiomeric excesses around 50%.

*Ni*FMO has been shown to be relatively thermostable. Therefore, some of the sulfoxidations performed at 30 °C were further tested at 45 °C, as shown in Table 2. For all the substrates, the enzyme retained the enantioselectivity while showing a higher activity at this temperature, as it was possible to recover the optically active sulfoxides with higher conversions. The further increase of the temperature to 60 °C in the sulfoxidation of **11a** led to enzyme deactivation (entry 4), and the recovery of (*S*)-**11b** with lower conversions and optical purities as compared to 45 °C.

### 2.2. Performance of mFMO Mutants in Enzymatic Sulfoxidations

**Figure 1:**
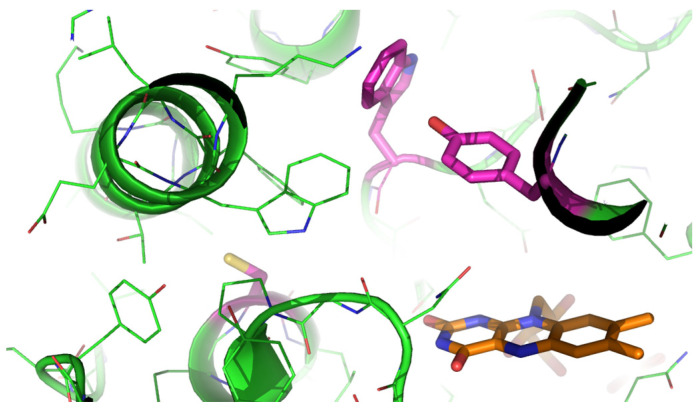
Active site of *m*FMO with the mutated residues (Cys78, Tyr207 and Trp319) highlighted in magenta and the flavin cofactor in orange (PDB:2VQ7).

Given that the utilization of *m*FMO resulted in higher conversions for most substrates in the tested sulfoxidation processes, as compared to *Ni*FMO, a series of *m*FMO mutants was generated and purified to assess their performance with sulfides. Previously, a few structure-inspired mutants had been developed, altering the substrate binding pocket: the single mutants C78I-*m*FMO and W319A-*m*FMO, as well as the triple mutant C78I/Y207W/W319A-*m*FMO. These three mutant enzymes had previously demonstrated enhanced activity for indole oxidation [29]. The mutations center around the predicted substrate binding pocket, which is next to the redox-active moiety of the bound flavin cofactor (Figure 1). Each residue forms a distinct part of the part of the surface of this pocket. This explains why each the use of different mutations can result in different effects on substrate binding and positioning.

The results obtained with the single mutants and the triple mutant are presented in Table 3 and compared with those obtained using wild-type *m*FMO. The biooxidation of alkyl phenyl sulfides **1–2a** and **4a** yielded the most selective products when utilizing both the W319A mutant and the triple mutant, achieving (*S*)-**1b** with optical purities around 63% and (*S*)-**2b** with enantiomeric excess of 94% (W319A) and 83% (triple mutant). Regarding the propyl derivative, W319A exhibited the best performance, enabling the recovery of 37% of (S)-**4b** with a 77% enantiomeric excess. No oxidation was observed for phenyl vinyl sulfide (**3a**) for either of the biocatalysts. The biooxidation of cyclopropyl phenyl sulfide (**5a**) resulted in the formation of (S)-**5b** with conversions around 30–40%, with the triple mutant achieving the highest optical purity (*ee* = 35%). Alkyl phenyl sulfides containing heteroatoms in the alkyl group were not as favorable as substrates for the enzymes in terms of selectivity, with optical purities of only around 30% obtained with the wild-type variant. The triple mutant was unable to oxidize either **6a** or **7a**, while W319A showed no activity for **7a**. C78I yielded the highest conversions in the formation of (*S*)-**6b** and (*S*)-**7b**, but with very low enantioselectivities.

When examining a series of thioanisole derivatives containing different substituents on the aromatic ring (both electron-withdrawing and electron-donating), both wild type and C78I mutant exhibited the best results in terms of activity and selectivity, contrasting with the lower performance of W319A and the triple mutant for this type of compound. For some of these substrates, including *p*-methoxy (**9a**), *m*-chloro (**12a**), and *p*-cyano (**15a**) derivatives, C78I led to an increase in system selectivity compared to the wild-type biocatalyst. This is particularly evident for the preparation of (*S*)-**12b**, which can be recovered with 70% *ee* when employing C78I, compared to the 15% *ee* achieved in the oxidation catalyzed by the wild-type enzyme. The optimal performance is obtained in the sulfoxidation of *p*-methyl (**10a**), *p*-chloro (**11a**), and *p*-bromo (**14a**) derivatives, with enantiomeric excesses exceeding 80% found when using either the wild-type enzyme or the C78I mutant. When analyzing the effect of chlorine position on the aromatic ring, C78I mutant showed a similar pattern compared with the wild-type enzyme. Once again, the *p*-chloro derivative was the best substrate (77%, 95% *ee*), with a good result for the meta-derivative and a lower optical purity, and especially as to conversion for the preparation of (*S*)-**13b**. W319A showed good conversion in the oxidation of *p*-bromothioanisole and was able to oxidize m-chlorothioanisole with 65% ee and 47%, but in general, moderate conversions and low optical purities were obtained. The triple mutant achieved the best performance in recovering methyl *p*-methoxyphenyl sulfoxide (*S*)-**9b** (56%, 65% *ee*) and methyl *p*-chlorophenyl sulfoxide (S)-**11b** (43%, 61% *ee*), whereas for the rest of the sulfides modest results were obtained. The sulfoxidation of a sulfide containing a strong electron-withdrawing group such as *p*-cyanothioanisole (**15a**) led to low or moderate optical purities for all tested *m*FMOs.

C78I increased significantly the conversion of sulfide **16a** compared with the wild-type enzyme, thus obtaining a 37% of (*S*)-**16b** with 35% *ee* after 24 h. No oxidation for this substrate was observed for W319A, whereas the triple mutant afforded the desired sulfoxide with the same low conversion of the wild-type enzyme (8%), but slightly lower optical purity (27% *ee*). None of the *m*FMO mutants were able to perform the oxidation of bulkier substrates, oxidizing neither benzyl phenyl sulfide (**17a**) nor pyrmetazole (**18a**), even when working with lower substrate concentrations (5 mM) and longer reaction times (72 h).

Benzylic substrates were also tested, revealing a reversal of enantiopreference, depending on the biocatalyst employed. Biooxidations catalyzed by C78I and the triple mutant afforded (*R*)-**19b** and (*R*)-**20b**. Contrastingly, the wild-type enzyme and the W319A mutant led to the (S)-sulfoxides. The W319A mutant produced the highest enantiomeric excesses for (*S*)-**19b** (*ee* = 43%) and (*S*)-**20b** (*ee* = 35%). In contrast, the (*R*)-enantiomers were achieved with low optical purities (10–20% *ee*). The highest conversions were observed in the sulfoxidations of **20a** catalyzed by both C78I and the triple mutant, yielding around 70% of (*R*)-**20b** after 24 h. The methyl derivative was obtained in lower conversions for both (*R*)-selective biocatalysts.

To test the effects of temperature on the biocatalysts’ properties, the oxidation of ethyl phenyl sulfide (**2a**) was carried out at 45 °C and pH 9.0 with all the monooxygenases. All four biocatalysts showed lower conversions and optical purities in the formation of the corresponding chiral sulfoxides compared to the results at 25 °C, indicating a negative influence of this parameter on the biocatalytic properties of both the wild-type and mutant enzymes (see Supporting Information).

**Figure 2:**
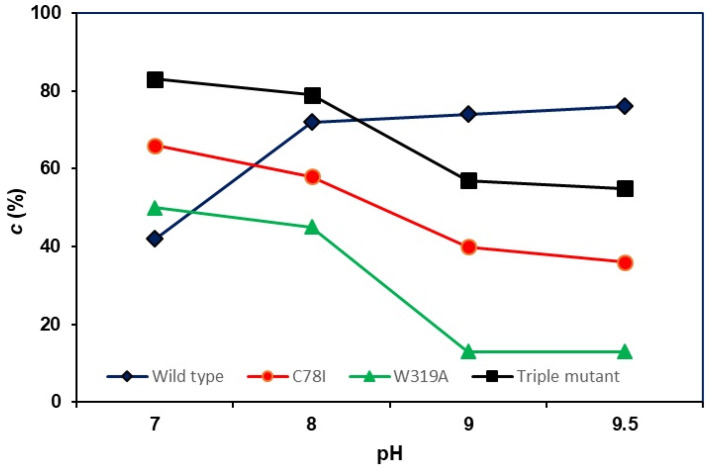
Effect of the pH on the conversion of the thioanisole sulfoxidation catalyzed by wild-type *m*FMO and its mutants.

In biocatalyzed processes, the protonation state of the catalytic residues is essential for achieving efficient reactions. Therefore, the effect of pH on the biocatalytic properties of wild-type *m*FMO and its three mutants was studied. Previous research has shown that pH modification greatly influences the activity of monooxygenases, such as BVMO from *Thermobifida fusca* (PAMO) [32]. In this study, no effect of pH (ranging from 7.0 to 9.5) on enzyme selectivity was observed when oxidizing thioanisole (**1a**) as a model substrate (see Supporting Information, Figure 2). However, different behaviors in enzymatic activity were noted. For wild-type *m*FMO, an increase in conversion was observed when moving from pH 7.0 to 8.0, with a slightly higher conversion seen at pH 9.5, achieving a 76% conversion of (S)-**1b** after 24 h. In contrast, the mutants C78I, W319A, and the triple mutant showed the highest conversions at pH 7.0, with a decrease in activity at more basic pHs, especially at values higher than 9.0.

**Figure 3:**
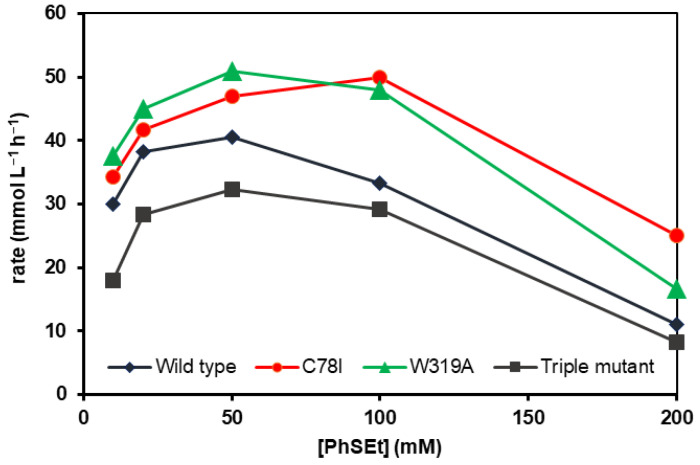
Effect of the ethyl phenyl sulfide (**2a**) concentration in the reaction rate (expressed as mmoles of sulfide consumed per hour and liter) of the sulfoxidation catalyzed by wild-type *m*FMO and its mutants.

The effects of **2a** concentration on the conversion and the enantioselectivity of the enzymatic sulfoxidation catalyzed by the mFMO biocatalysts were also analyzed (Figure 3). To compare the conversions obtained at different times (24–72 h), the reaction rate, expressed as the millimoles of **2a** consumed per hour per liter, was defined. Up to concentrations of 50 mM, the best results were achieved with W319A. However, from 100 to 200 mM, C78I showed better performance. Wild-type *m*FMO, W319A and the triple mutant exhibited similar behavior. The reaction rate increased with rising **2a** concentrations, reaching a maximum around 50 mM (40.6 mmol L^−1^ h^−1^ for the wild-type enzyme; 51.0 mmol L^−1^ h^−1^ for W319A; and 32.3 mmol L^−1^ h^−1^ for the triple mutant). Beyond this concentration, the reaction rate decreased. C78I displayed a slightly different trend, with the highest reaction rate observed at 100 mM 2a (50.0 mmol L^−1^ h^−1^) and a lower but still good rate at a substrate concentration of 200 mM (25.0 mmol L^−1^ h^−1^). For all biocatalysts tested, no changes in selectivity were observed with increasing substrate concentration, as each enzyme achieved similar enantiomeric excesses under all conditions analyzed (Supporting Information).

As W319A mutant led to the best performance in the oxidation of ethyl phenyl sulfide, this process was scaled up to multimilligram scale, thus oxidizing 28.0 mg of **2a** in buffer Tris/HCl pH 9.0 at room temperature. After 50 h, (*S*)-ethyl phenyl sulfoxide (**2b**) could be obtained with 58% yield and 93% enantiomeric excess.

## 3. Materials and Methods

### 3.1. Materials and Methods

Purified PTDH-fused *Methylophaga* sp. FMO (*m*FMO) and its mutants were obtained as previously described [28,29]. Purified *Nitrincola lacisaponensis* FMO (*Ni*FMO) was achieved following the procedure described in [30]. Sodium phosphite dibasic pentahydrate, starting sulfides **1a**, **3-5a**, **7–8a**, **14a**, racemic sulfoxide (±)-**1b** and omeprazole (±)-**18b** were purchased from Sigma-Aldrich (Steinheim, Germany). Sulfides **2a**, **6a**, **9a**, **12–13a**, **18a** and **19a** were obtained from TCI Europe (Zwijndrecht, Belgium). Sulfide **11a** was purchased from Acros Organics (Geel, Belgium). NADPH and compounds **10a** and **15–17a** were purchased from Alfa Aesar (Karlsruhe, Germany). Sulfide **13a** was prepared as previously described, employing ethyl iodide and benzyl mercaptan in basic medium [26]. Racemic sulfoxides (±)-**2–17b** and (±)-**19**,**20b** were prepared by oxidation of the corresponding sulfides, employing hydrogen peroxide in methanol. Unless otherwise stated, analytical-grade solvents and commercially available reagents were used without further purification.

GC/MS analyses were performed with a GC Hewlett Packard 7890 Series II equipped with a Hewlett Packard 5973 chromatograph MS (Agilent Technologies, Santa Clara, CA, USA) using a HP-5MS cross-linked methyl siloxane column (30 m × 0.25 mm × 0.25 μm, 1.0 bar N_2_). To monitor levels of conversion, substrates and products were quantified by the use of calibration curves. HPLC analyses were carried out with a Thermo-Fischer UltiMate chromatograph equipped with a Thermo UltiMate detector (Thermo-Fischer, Whaltham, MA, USA). To determine the level of conversion of the esomeprazole sulfide (**18a**) oxidation, a calibration curve using HPLC was employed. Absolute configuration of the chiral sulfoxides was established by comparison with the data described in [28,33].

### 3.2. General Procedure for the FMO-Catalyzed Sulfoxidation of Sulfides ***1–20a***

Unless otherwise stated, prochiral sulfides **1–20a** (5–10 mM) were dissolved in 1.0 mL Tris/HCl 50 mM (pH 9.0) containing DMSO (10 µL), NADPH (0.2 mM), sodium phosphite (10 mM) and the corresponding FMO (1.0 μM). Reactions were stirred at room temperature and 220 rpm for the times established. Once finished, the reactions were extracted with EtOAc (2 × 0.5 mL) and dried onto Na_2_SO_4_, and the samples were directly analyzed by GC/MS and HPLC to determine the level of conversion as well as the enantiomeric excesses of the chiral sulfoxides (*R*)- or (*S*)-**1–20b**. 

### 3.3. W319A Biocatalyzed Synthesis of (S)-Ethyl Phenyl Sulfoxide (***2b***) at Multimilligram Scale

Ethyl phenyl sulfide (**2a**, 28.0 mg, 20 mM) was dissolved in a Tris/HCl 50 mM solution (pH 9.0) containing DMSO (100 µL), NADPH (0.2 mM), sodium phosphite (10 mM) and W319A (1.0 μM) up to a final volume of 10.0 mL. The reaction was stirred at room temperature and 220 rpm for 50 h. Once finished, the crude reaction was extracted with EtOAc (2 × 5 mL) and dried onto Na_2_SO_4_, and the solvent was removed under reduced pressure to yield 11.9 mg of crude (85% conversion). The mixture was purified by column chromatography using *n*-hexane/ethyl acetate 7:3 as eluent to afford 17.8 mg of (*S*)-**2b** (58% yield) as a yellow pale oil with a 93% enantiomeric excess. 

## 4. Conclusions

The studied bacterial flavin-containing monooxygenases *m*FMO and *Ni*FMO were found to be very useful biocatalysts when employed to produce optically active sulfoxides. *Ni*FMO can convert prochiral sulfides with moderate results. The best results were obtained in the preparation of *p*-methyl-, *p*-chloro- and *p*-bromophenyl methyl sulfoxides. Results with this biocatalyst can be further improved by working at 45 °C, which results in higher conversions while retaining the enantioselectivity. Overall, *m*FMO was found to be a superior biocatalyst for sulfoxidations; this motivated us to test some selected *m*FMO mutants. The use of the C78I mFMO mutant led to an increase in the selectivity of the final products when oxidizing aryl derivatives of methyl phenyl sulfides. Some of the corresponding (*S*)-sulfoxides could be obtained with optical purities and good conversions. W319A mFMO and the triple mFMO mutant are better oxidative biocatalysts for oxidizing alkyl phenyl sulfides up to the propyl group. These two biocatalysts allow production of the final compounds with moderate-to-high enantiomeric excesses and activities. Depending on the biocatalyst employed, it was possible to achieve the alkyl benzyl sulfoxides with opposite enantiopreference. The best results were obtained concerning formation of the (*S*)-enantiomers by using the wild-type or W319A mFMO variants. Both C78I mFMO and the triple mutant afforded (*R*)-sulfoxides with low selectivity. These findings highlight the potential for engineering FMOs to fine-tune substrate specificity and enantioselectivity.

## Figures and Tables

**Table 1:** Biocatalysed sulfoxidations catalyzed by *m*FMO and *Ni*FMO ^1^.

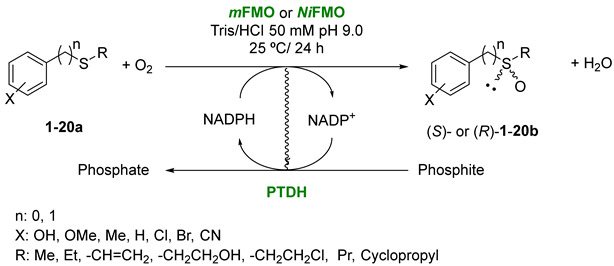					
		*m*FMO		*Ni*FMO	
Entry	Sulfide	Conv. (%) ^2^	*ee* (%) ^3^	Conv. (%) ^2^	*ee* (%) ^3^
Entry 1 ^4^	PhSMe (**1a**)	90	35 (*S*)	17	9 (*R*)
Entry 2 ^4^	PhSEt (**2a**)	18	79 (*S*)	25	57 (*S*)
Entry 3	PhSCH=CH_2_ (**3a**)	≤3	n.d.	≤3	n.d.
Entry 4	PhSPr (**4a**)	12	64 (*S*)	10	55 (*S*)
Entry 5	PhSCyclopropyl (**5a**)	39	19 (*S*)	≤3	n.d.
Entry 6	PhSCH_2_CH_2_OH (**6a**)	78	28 (*S*)	43	41 (*S*)
Entry 7	PhSCH_2_CH_2_Cl (**7a**)	70	33 (*S*)	40	49 (*S*)
Entry 8	*p*-HO-PhSMe (**8a**)	56	72 (*S*)	8	17 (*S*)
Entry 9 ^4^	*p*-MeO-PhSMe (**9a**)	78	70 (*S*)	12	29 (*S*)
Entry 10 ^4^	*p*-Me-PhSMe (**10a**)	66	92 (*S*)	97	76 (*S*)
Entry 11 ^4^	*p*-Cl-PhSMe (**11a**)	80	95 (*S*)	32	83 (*S*)
Entry 12 ^4^	*m*-Cl-PhSMe (**12a**)	69	15 (*S*)	31	12 (*S*)
Entry 13 ^4^	*o*-Cl-PhSMe (**13a**)	20	64 (*S*)	23	15 (*S*)
Entry 14	*p*-Br-PhSMe (**14a**)	75	85 (*S*)	22	75 (*S*)
Entry 15 ^4^	*p*-NC-PhSMe (**15a**)	50	22 (*S*)	19	32 (*S*)
Entry 16 ^5^	NaphSMe (**16a**)	8	39 (*S*)	≤3	n.d.
Entry 17 ^5^	PhSBn (**17a**)	≤3	n.d.	≤3	n.d.
Entry 18 ^5^	Pyrmetazole (**18a**)	≤3	n.d.	≤3	n.d.
Entry 19 ^4^	BnSMe (**19a**)	85	17 *(S*)	38	51 (*R*)
Entry 20 ^4^	BnSEt (**20a**)	51	15 (*S*)	12	41 (*R*)

^1^ For reaction details, see Materials and Methods. ^2^ Determined by GC/MS. ^3^ Determined by HPLC. ^4^ Previously reported for *m*FMO by [28]. ^5^ Reactions performed with 5 mM sulfide concentration for 36 h. n.d. not determined.

**Table 2:** Temperature effect on *Ni*FMO-biocatalyzed sulfoxidations.

Entry	Sulfide	T (°C)	Time (h)	Conv. (%) ^1^	*ee* (%) ^2^
Entry 1	PhSEt (**2a**)	45	24	40	55 (*S*)
Entry 2	*p*-Me-PhSMe (**10a**)	45	14	97	75 (*S*)
Entry 3	*p*-Cl-PhSMe (**11a**)	45	24	61	84 (*S*)
Entry 4	*p*-Cl-PhSMe (**11a**)	60	24	15	27 (*S*)
Entry 5	*p*-Br-PhSMe (**14a**)	45	24	35	72 (*S*)
Entry 6	BnSMe (**19a**)	45	24	54	50 (*S*)

^1^ Determined by GC/MS. ^2^ Measured by HPLC.

**Table 3:** Performance of *m*FMO wild type and mutants on sulfide oxidation ^1^.

Sulfide	Wild Type	C78I	W319A	Triple Mutant
PhSMe (**1a**)	90% 35% (*S*)	40% 10% (*S*)	13% 64% (*S*)	57% 63% (*S*)
PhSEt (**2a**)	72% 75% (*S)*	82% 75% (*S)*	90% 94% (*S)*	43% 83% (*S)*
PhSPr (**4a**)	32% 64% (*S*)	25% 60% (*S*)	37% 77% (*S*)	15% 65% (*S*)
PhSCyclopropyl (**5a**)	39% 19% (*S*)	43% 15% (*S*)	41% 17% (*S*)	30% 35% (*S*)
PhSCH_2_CH_2_OH (**6a**)	78% 28% (*S*)	81% 5% (*S*)	17% 9% (*S*)	≤3% n.d.
PhSCH_2_CH_2_Cl (**7a**)	70% 33% (*S*)	61% 12% (*S*)	≤3% n.d.	≤3% n.d.
*p*-HO-PhSMe (**8a**)	56% 72% (*S*)	33% 69% (S)	≤3% n.d.	28% 45% (S)
*p*-MeO-PhSMe (**9a**)	78% 70% (*S*)	43% 85% (*S*)	18% 30% (*S*)	56% 65% (*S*)
*p*-Me-PhSMe (**10a**)	66% 92% (*S*)	70% 90% (*S*)	33% 9% (*S*)	58% 63% (*S*)
*p*-Cl-PhSMe (**11a**)	80% 95% (*S*)	77% 95% (*S*)	66% 5% (*S*)	43% 61% (*S*)
*m*-Cl-PhSMe (**12a**)	69% 15% (*S*)	73% 70% (*S*)	47% 65% (*S*)	49% 27% (*S*)
*o*-Cl-PhSMe (**13a**)	20% 64% (*S*)	24% 62% (*S*)	27% 15% (*S*)	8% 22% (*S*)
*p*-Br-PhSMe (**14a**)	75% 85% (*S*)	67% 83% (*S*)	80% 7% (*S*)	37% 59% (*S*)
*p*-NC-PhSMe (**15a**)	50% 22% (*S*)	37% 41% (*S*)	63% 7% (*S*)	15% 37% (*S*)
NaphSMe (**16a**)	8% 39% (*S*)	37% 35% (*S*)	≤3% n.d.	8% 27% (*S*)
BnSMe (**19a**)	58% 14% (*S*)	31% 17% (*R*)	35% 43% (*S*)	20% 20% (*R*)
BnSEt (**20a**)	51% 15% (*S)*	69% 12% (*R)*	76% 35% (*S)*	69% 10% (*R)*

^1^ Conversions were determined by GC/MS and enantiomeric excesses were determined by chiral HPLC. n.d.: not determined.

## Data Availability

The original contributions presented in the study are included in the article (and supplementary material), further inquiries can be directed to the corresponding authors.

## Supplementary Materials

The following supporting information can be downloaded at: https://www.mdpi.com/article/10.3390/molecules29153474/s1, Table S1: *m*FMO mutants catalyzed sulfoxidations at 45 °C. Table S2: Effect of pH in the biocatalyzed oxidations of **2a** performed by wild-type mFMO and its mutants. Table S3: Results of the **2a** concentration effect in the sulfoxidation catalyzed by *m*FMO mutants. Table S4: Retention times at GC/MS analyses in the biooxidations of prochiral sulfides **1-17a** and **19-21a**. Table S5: Determination of enantiomeric excesses by HPLC analyses.

## References

[1] Wojaczynska E., Wojaczynski J. (2023). Sulfoxides in medicine. Curr. Opin. Chem. Biol..

[2] Bentley R. (2005). Role of sulfur chirality in the chemical processes of biology. Chem. Soc. Rev..

[3] Han J., Soloshonok V.A., Klika K.D., Drabowicz J., Wzorek A. (2018). Chiral sulfoxides: Advances in asymmetric synthesis and problems with the accurate determination of the stereochemical outcome. Chem. Soc. Rev..

[4] Salom-Reig X., Bauder C. (2020). Recent Applications in the Use of Sulfoxides as Chiral Auxiliaries for the Asymmetric Synthesis of Natural and Biologically Active Products. Synthesis.

[5] Trost B.M., Rao M. (2015). Development of chiral sulfoxide ligands for asymmetric catalysis. Angew. Chem. Int. Ed..

[6] Wojaczynskak E., Wojaczynski J. (2020). Modern stereoselective synthesis of chiral sulfinyl compounds. Chem. Rev..

[7] Yang M.M., Wang S., Dong Z.B. (2022). Recent advances for chiral sulfoxides in asymmetric catalysis. Synthesis.

[8] Devine P.N., Howard R.M., Kumar R., Thompson M.P., Truppo M.D., Turner N.J. (2018). Extending the application of biocatalysis to meet the challenges of drug development. Nat. Chem. Rev..

[9] Wu S., Snajdrova R., Moore J.C., Baldenius K., Bornscheuer U.T. (2021). Biocatalysis: Enzymatic Synthesis for Industrial Applications. Angew. Chem. Int. Ed..

[10] Simíc S., Zukíc E., Schmermund L., Faber K., Winkler C.K., Kroutil W. (2022). Shortening synthetic routes to small molecule Active Pharmaceutical Ingredients employing biocatalytic methods. Chem. Rev..

[11] Dong J.J., Fernández-Fueyo E., Hollmann F., Paul C.E., Pesic M., Schmidt S., Wang Y., Younes S., Zhang W. (2018). Biocatalytic oxidation reactions: A chemist’s perspective. Angew. Chem. Int. Ed..

[12] Maczka W., Winska K., Grabarczyk M. (2018). Biotechnological Methods of Sulfoxidation: Yesterday, Today, Tomorrow. Catalysts.

[13] Bassanini I., Ferrandi E.E., Vanoni M., Ottolina G., Riva S., Crotti M., Brenna E., Monti D. (2017). Peroxygenase-catalyzed enantioselective sulfoxidations. Eur. J. Org. Chem..

[14] Linde D., Cañella M., Coscolín C., Davó-Siguero I., Romero A., Lucas F., Ruiz-Dueñas F.J., Guallar V., Martínez A.T. (2016). Asymmetric sulfoxidation by engineering the heme pocket of a dye-decolorizing peroxidase. Catal. Sci. Technol..

[15] Sanfilippo C., Cernuto F., Patti A. (2023). Expanding the Use of Peroxygenase from Oat Flour in Organic Synthesis: Enantioselective Oxidation of Sulfides. Int. J. Mol. Sci..

[16] Boyd D.R., Sharma N.D., Stevenson P.J., Hoering P., Allen C.C.R., Dansette P.M. (2022). Monooxygenase- and Dioxygenase-Catalyzed Oxidative Dearomatization of Thiophenes by Sulfoxidation, *cis*-Dihydroxylation and Epoxidation. Int. J. Mol. Sci..

[17] Willrodt C., Gröning J.A.D., Nerke P., Koch R., Scholtissek A., Heine T., Schmid A., Bühler B., Tischler D. (2020). Highly Efficient Access to (S)-Sulfoxides Utilizing a Promiscuous Flavoprotein Monooxygenase in a Whole-Cell Biocatalyst Format. ChemCatChem.

[18] Liu F., Shou C., Geng Q., Zhao C., Xu J., Yu H. (2020). A Baeyer-Villiger monooxygenase from *Cupriavidus basilensis* catalyzes asymmetric synthesis of (*R*)-lansoprazole and other pharmaco-sulfoxides. Appl. Microbiol. Biotechnol..

[19] Wang J.-B., Huang Q., Peng W., Wu P., Yu D., Chen B., Wang B., Reetz M.T. (2020). P450-BM3-Catalyzed Sulfoxidation versus Hydroxylation: A Common or Two Different Catalytically Active Species?. J. Am. Chem. Soc..

[20] Boredwick S., Beier A., Balke K., Bornscheuer U.T. (2018). Baeyer-Villiger monooxygenases from *Yarrowia lipolytica* catalyze preferentially sulfoxidations. Enzyme Microb. Technol..

[21] Romero E., Gómez Castellanos J.R., Gadda G., Fraaije M.W., Mattevi A. (2018). Same Substrate, Many Reactions: Oxygen Activation in Flavoenzymes. Chem. Rev..

[22] Catucci C., Gao C., Sadeghi S.J., Gilardi G. (2017). Chemical applications of Class B flavoprotein monooxygenases. Rend. Fis. Acc. Lincei.

[23] Hamman M.A., Haehner-Daniels B.D., Wrighton S.A., Rettie A.E., Hall S.D. (2000). Stereoselective sulfoxidation of sulindac sulfide by flavin-containing monooxygenases. Comparison of human liver and kidney microsomes and mammalian enzymes. Biochem. Pharmacol..

[24] Phillips I.R., Sheppard E.A. (2019). Endogenous Roles of Mammalian Flavin-Containing Monooxygenases. Catalysts.

[25] Nicol C.R., Mascotti M.L. (2023). Investigating the biochemical signatures and physiological roles of the FMO family using molecular phylogeny. BBA Adv..

[26] Choi H.S., Kim J.K., Cho E.H., Kim Y.C., Kim J.I., Kim S.W. (2003). A novel flavin-containing monooxygenase from *Methylophaga sp*. strain SK1 and its indigo synthesis in *Escherichia coli*. Biochem. Biophys. Res. Commun..

[27] Alfieri A., Malito E., Orru R., Fraaije M.W., Mattevi A. (2008). Revealing the moonlighting role of NADP in the structure of a flavin-containing monooxygenase. Proc. Natl. Acad. Sci. USA.

[28] Rioz-Martínez A., Kopacz M., de Gonzalo G., Torres Pazmiño D.E., Gotor V., Fraaije M.W. (2010). Exploring the biocatalytic scope of a bacterial flavin-containing monooxygenase. Org. Biomol. Chem..

[29] Lončar N., van Beek H.L., Fraaije M.W. (2019). Structure-based redesign of a self-sufficient flavin-containing monooxygenase towards indigo production. Int. J. Mol. Sci..

[30] Lončar N., Fiorentini F., Bailleul G., Savino S., Romero E., Mattevi A., Fraaije M.W. (2019). Characterization of a thermostable flavin-containing monooxygenase from *Nitrincola lacisaponensis* (*Ni*FMO). Appl. Microbiol. Biotechnol..

[31] Torres Pazmiño D.E., Snajdrova R., Baas B.-J., Ghobrial M., Mihovilovic M.D., Fraaije M.W. (2008). Self-sufficient Baeyer-Villiger monooxygenases: Effective Coenzyme Regeneration for Biooxygenation by Fusion Engineering. Angew. Chem. Int. Ed..

[32] Zambianchi F., Fraaije M.W., Carrea G., de Gonzalo G., Rodríguez C., Gotor V., Ottolina G. (2007). Titration and assignment of residues that regulate the enantioselectivity of phenylacetone monooxygenase. Adv. Synth. Catal..

[33] de Gonzalo G., Torres Pazmiño D.E., Ottolina G., Fraaije M.W., Carrea G. (2005). Oxidations catalyzed by phenylacetone monooxygenase from *Thermobifida fusca*. Tetrahedron Asymmetry.

