# Supplementary material for: Asymmetric Sulfoxidations Catalyzed by Bacterial Flavin-Containing Monooxygenases
Source: Molecules. 2024 Jul 25;29(15):3474. doi: 10.3390/molecules29153474 (PMC11313838; doi:10.3390/molecules29153474)
Supplement: Supplementary file 1 [file molecules-29-03474-s001.zip › molecules-3096787-supplementary.pdf]

Electronic Supporting Information

Asymmetric sulfoxidations catalyzed by bacterial flavin-containing monooxygenases

Gonzalo de Gonzalo,[1,*] Juan M. Coto-Cid,[1] Nikola Lončar[2] and Marco W. Fraaije[3]

[1] Departamento de Química Orgánica, Universidad de Sevilla, c/ Profesor García González 1, 41012, Sevilla, Spain. Mail: gdegonzalo@us.es. Phone: +34 503634345

[2] Gecco Biotech B.V., Nijenborgh 4, 9747AG, Groningen, The Netherlands.

[3] Groningen Biomolecular Sciences and Biotechnology Institute, University of Groningen, Nijenborgh 4, 9747, AG Groningen, The Netherlands.

1. Results.
2. GC analyses.
3. HPLC analyses.

1. Results

1.1. Effect of the temperature in the biocatalysed sulfoxidation of prochiral sulfides in presence of wild type mFMO and the C78I, W319A and triple mutants.

Sulfides **1a, 2a, 6a** or **10a** were oxidised by *m*FMO mutants at 10 mM concentration when working in Tris/HCl 50 mM pH 9.0 containing 1% v/v DMSO. Reactions were carried out at 45ºC and 220 rpm for 24 hours. Results are summarized in Table S1.

Table S1. *m*FMO mutants catalysed sulfoxidation of prochiral sulfides at 45ºC.

Entry	Biocatalyst	Sulfide	Conv. (%) [1]	*ee* (%) [2]
1	Wild type	**10a**	50	83
2	C78I	**6a**	37	73
2	W319A	**1a**	10	50
3	Triple	**2a**	27	65

[1] Measured by GC/MS.

[2] Determined by chiral HPLC.

1.2. Effect of the pH in the biocatalysed sulfoxidation of thioanisole in presence of wild type mFMO and the C78I, W319A and triple mutants.

Thioanisole (**1a**) was oxidised by *m*FMO wild type and mutants at 10 mM concentration when working in Tris/HCl 50 mM pH 7.0-9.5 containing 1% v/v DMSO. Reactions were carried out at 25ºC and 220 rpm for 24 hours. Results are summarized in Table S2.

Table S2. *m*FMO-catalysed sulfoxidation of **1a** at different pHs.

Entry	Biocatalyst	pH	Conv. (%) [1]	*ee* (%) [2]
1	Wild type	7.0	42	33

2	Wild type	8.0	72	35
3	Wild type	9.0	74	35
4	Wild type	9.5	76	37
5	C78I	7.0	66	13
6	C78I	8.0	58	12
7	C78I	9.0	40	10
8	C78I	9.5	36	10
9	W319A	7.0	50	67
10	W319A	8.0	45	67
11	W319A	9.0	13	64
12	W319A	9.5	13	65
13	Triple	7.0	83	63
14	Triple	8.0	79	65
15	Triple	9.0	57	63
16	Triple	9.5	55	60

n.d. Not determined.

[1] Measured by GC/MS.

[2] Determined by chiral HPLC.

1.3. *Effect of ethyl phenyl sulfide (**2a**) concentration in the sulfoxidations catalysed by C78I-mFMO and W319A-mFMO.*

Ethyl phenyl sulfide (**2a**) was oxidised by C78I or W319A-*m*FMO at different concentrations when working in Tris/HCl 50 mM pH 9.0 containing 1% v/v DMSO. Reactions were carried out at 25ºC and 220 rpm for 24-48 hours. Results are summarized in Table S3.

Table S3. *m*FMO C78I and W319A mutants catalysed sulfoxidation of **2a** at different concentrations.

Entry	Biocatalyst	[2a] (mM)	time (h)	Conv. (%) [1]	Reaction rate (mmol/ L h)	*ee* (%) [2]
1	Wild type	10	24	72	30.0	75
2	Wild type	20	24	46	38.3	76
3	Wild type	50	48	39	40.6	75
4	Wild type	100	48	14	29.2	73
5	Wild type	200	72	8	13.9	72
6	C78I	10	24	82	34.2	75
7	C78I	20	24	50	41.7	73
8	C78I	50	48	45	46.9	76
9	C78I	100	48	34	50.0	73
10	C78I	200	72	9	25.0	73
11	W319A	10	24	90	37.5	94
12	W319A	20	24	65	54.2	94
13	W319A	50	48	49	51.0	92
14	W319A	100	48	23	47.9	91
15	W319A	200	72	6	16.7	90
16	Triple mutant	10	24	43	18.9	83
17	Triple mutant	20	24	34	28.3	80

18	Triple mutant	50	48	31	32.3	81
19	Triple mutant	100	48	14	29.2	83
20	Triple mutant	200	72	3	8.3	82

[1] Measured by GC/MS.

[2] Determined by chiral HPLC.

2. Gas chromatography analyses

GC/MS analysis for compounds **1–17** and **19-20** were performed on a GC Agilent 8890 GC System equipped with an Agilent 5977B GC/MSD Detector. Separations were carried out in a HP-5 cross-linked methyl siloxane column (30 m × 0.32 mm × 0.25 μm, 1.0 bar N_2). The injector temperature was 225 °C and the detector temperature was 250 °C. The temperature program performed for all the substrates was: 50ºC/ 5 min/ 10ºC/min/ 200ºC/ 2 min. To monitor levels of conversion, substrates and products were quantified by use of calibration curves.

Table S4. Retention times at GC/MS analyses in the biooxidations of prochiral sulfides **1-17a** and **19-20a**.

Compound	t_R **a** (min)	t_R **b** (min)
**1**	11.0	15.1
**2**	12.2	16.2
**3**	11.9	15.8
**4**	13.0	17.3
**5**	13.3	17.2
**6**	15.7	18.6
**7**	15.4	18.7
**5**	14.9	18.6
**6**	12.7	16.5
**7**	14.1	17.1
**8**	14.1	17.0
**9**	14.9	18.6
**10**	12.7	16.5
**11**	14.2	17.1
**12**	14.1	17.2
**13**	14.1	17.1
**14**	15.4	18.4
**15**	16.5	18.9
**16**	18.9	21.8
**17**	19.5	22.8
**19**	12.5	17.0
**20**	13.4	18.1

3. HPLC analyses

For the determination of the optical purities of optically active sulfoxides **1-17b** and **19-20b**, the following columns were employed at 30ºC: column A: Chiralcel OD (0.46 cm x 25 cm), column B: Chiralpak IC (0.46 cm x 25 cm), and column C: Chiralcel OJ-H (0.46 cm x 25 cm), all from Daicel.

Table S5. Determination of enantiomeric excesses by HPLC analyses.

**Sulfoxide**	**Column**	**Flow (mL min^{-1})**	**Eluent**[a]	**Retention times (min)**
**1b**	A	1.0	*n*-hexane-IPA 9:1	10.2 (*R*); 12.0 (*S*)
**2b**	A	1.0	*n*-hexane-IPA 95:5	12.9 (*R*); 16.5 (*S*)
**3b**	A	1.0	*n*-hexane-IPA 9:1	16.3 (*R*); 18.5 (*S*)
**4b**	A	1.0	*n*-hexane-IPA 9:1	13.4 (*S*); 17.5 (*R*)
**5b**	A	1.0	*n*-hexane-IPA 9:1	9.5 (*R*); 11.9 (*S*)
**6b**	A	1.0	*n*-hexane-IPA 9:1	11.5 (*R*); 13.7 (*S*)
**7b**	A	1.0	*n*-hexane-IPA 95:5	19.1 (*R*); 20.7 (*S*)
**8b**	B	1.0	*n*-hexane-IPA 7:3	13.1 (S); 13.9 (R)
**9b**	A	1.0	*n*-hexane-IPA 9:1	14.1 (*R*); 15.2 (*S*)
**10b**	A	1.0	*n*-hexane-IPA 95:5	14.9 (*R*); 16.8 (*S*)
**11b**	B	1.0	*n*-hexane-IPA 85:15	22.6 (*S*); 24.0 (*R*)
**12b**	B	1.0	*n*-hexane-IPA 8:2	15.9 (*S*); 17.5 (*R*)
**13b**	B	1.0	*n*-hexane-IPA 85:15	17.1 (*S*); 17.9 (*R*)
**14b**	B	1.0	*n*-hexane-IPA 8:2	17.9 (*S*); 19.0 (*R*)
**15b**	C	1.0	*n*-hexane-IPA 9:1	47.1 (*R*); 52.3 (*S*)
**16b**	A	1.0	*n*-hexane-IPA 95:5	26.1 (*R*); 29.0 (*S*)
**17b**	A	0.5	*n*-hexane-IPA 9:1	22.1 (*R*); 26.7 (*S*)
**19b**	A	1.0	*n*-hexane-IPA 9:1	17.0 (*R*); 18.7 (*S*)
**20b**	A	1.0	*n*-hexane-IPA 9:1	17.9 (*R*); 19.5 (*S*)

The conversion and optical purity of omeprazole **18b** was determined by HPLC employing a Chiralpak IA (0.46 cm x 25 cm) column from Daicel. Retention time for omeprazole sulfide (pyrmetazole) **18a** was 6.2 min, whereas for the sulfoxide **18b**: t_R (*R*)=16.2 min, and t_R (*S*) = 19.1 min.
